# Prognostic value of circulating tumor cells in patients with bladder cancer: A meta-analysis

**DOI:** 10.1371/journal.pone.0254433

**Published:** 2021-07-09

**Authors:** Hui Jiang, Xiujuan Gu, Zhihua Zuo, Gang Tian, Jinbo Liu

**Affiliations:** Department of Laboratory Medicine, The Affiliated Hospital of Southwest Medical University, Luzhou, China; Shuguang Hospital, CHINA

## Abstract

**Background:**

Circulating tumor cells (CTCs) have been considered diagnostic and prognostic biomarkers for urothelial cancer. However, the prognostic role of CTCs in bladder cancer (BC) remains controversial. Here, we conducted a meta-analysis to evaluate the prognostic significance of CTCs for patients with BC.

**Methods:**

All studies relevant to this topic were searched in the PubMed, Embase, and Web of Science databases. The hazard ratio (HR) and 95% confidence interval (95% CI) were set as effect measures. The outcomes were overall survival (OS), cancer-free survival (CSS), progression-free survival (PFS)/time to progression (TTP), and disease-free survival (DFS)/recurrence-free survival (RFS)/time to first recurrence (TFR). All analyses were conducted in STATA 15.1.

**Results:**

Eleven eligible studies comprising 1,062 patients with BC were included in this meta-analysis. Overall analyses showed that CTC-positive patients had poorer survival (OS: HR 3.88, 95% CI 2.52–5.96, *p* < 0.001; CSS: HR 3.89, 95% CI 2.15–7.04, *p* < 0.001) and more aggressive progression (PFS/TTP: HR 5.92, 95% CI 3.75–9.35, *p* < 0.001; DFS/RFS/TFR: HR 4.57, 95% CI 3.34–6.25, *p* < 0.001) than CTC-negative patients. Subgroup analyses according to the number of patients, detection method, positivity rate, and follow-up time revealed that the presence of CTCs predicted a high risk of mortality and disease progression in most subgroups.

**Conclusion:**

The meta-analysis confirmed that CTCs are a promising prognostic biomarker of poor survival and aggressive tumor progression for patients with BC.

**Prospero registration number:**

CRD42021224865.

## Introduction

Bladder cancer (BC) ranks as the tenth most frequently diagnosed cancer worldwide. In 2018, there were approximately 550,000 new cases and 200,000 deaths due to BC, 80% of which occurred in men [1]. BC is subdivided into two types according to the different treatment modes and prognoses: nonmuscle invasive bladder cancer (NMIBC) and muscle invasive bladder cancer (MIBC). NMIBC tends to relapse and requires long-term monitoring, while aggressive MIBC is prone to metastasis and is associated with a high rate of mortality [2, 3]. Treatments for NMIBC comprise endoscopic resection and adjuvant intravesical therapy. Instillation with bacillus Calmette-Guerin (BCG) is unfortunately ineffective in patients with high-risk NMIBC [4]. Current treatments of MIBC include radical cystectomy (RC) or trimodal therapy with maximal endoscopic resection, radiosensitizing chemotherapy, and radiation, which contribute to inhibition of tumor metastasis and reduction of disease mortality [5]. Despite these treatment measures, the overall therapeutic effects for BC are unsatisfactory, and the 5-year survival rate decreases with the increase in tumor stage, with the lowest survival rate of only 15% in patients with stage 4 tumors [6]. Therefore, identification of a risk assessment marker for predicting disease progression and outcome could be beneficial for treating patients with BC.

Circulating tumor cells (CTCs) are tumor cells released into the bloodstream from the primary tumor and participate in the process of metastasis and cancer recurrence [7, 8]. The prognostic role of CTCs has been reported in various types of cancers, such as breast [9], lung [10, 11], gastric [12–14], prostate [15] and colorectal [16, 17]. For patients with urothelial cancer (UC), two published meta-analyses have shown the diagnostic and prognostic value of the detection of CTCs [18, 19]. For patients with BC, although numerous cohort studies have investigated the correlation between the presence of CTCs and the survival of these patients, contrasting results have led to confusion and concerns regarding the clinical value of CTCs.

In the present meta-analysis, we included studies on the role of CTCs in BC and investigated the potential utility of CTCs in clinical practice. The prognostic outcomes were overall survival (OS), cancer-free survival (CSS), progression-free survival (PFS)/time to progression (TTP), and disease-free survival (DFS)/recurrence-free survival (RFS)/time to first recurrence (TFR).

## Materials and methods

### Registration

The protocol was registered on the Prospective Register of Systematic Reviews (PROSPERO); the registration number is CRD42021224865.

### Data source and search strategy

All relevant articles were searched through PubMed, Embase, and Web of Science in November 2020 and updated on 10 March 2021. The Medical Subject Heading (MeSH) terms and text words used for literature retrieval were as follows: (1) neoplastic cells, circulating or circulating tumor cells or CTCs; (2) urinary bladder neoplasms or bladder cancer or bladder transitional cell carcinoma or urothelial carcinoma of the bladder or urothelial cancer. The language was limited to English. The detailed search strategies of each database are displayed in S1 Text. In addition, the references of the identified studies were also searched for additional relevant documents.

### Inclusion and exclusion criteria

Two investigators (HJ and XJG) assessed whether each of the identified manuscripts was eligible. If necessary, disagreements were resolved by a third investigator (ZHZ). The inclusion criteria for our meta-analysis were as follows: (1) studies investigating the association between CTC status and the prognosis of BC patients; (2) studies providing HRs with 95% CIs or Kaplan-Meier curves; and (3) studies enrolling more than 20 patients.

The exclusion criteria were as follows: (1) review, letter, conference abstract or case report; and (2) samples not collected from peripheral blood.

### Data extraction

Two investigators (HJ and XJG) independently assessed the included studies and extracted the baseline characteristics (name of the first author, publication year, diagnosis, numbers of patients, detection methods, positivity rates, follow-up time, and outcomes). If necessary, disagreements were resolved by a third investigator (ZHZ). When there was more than one group in a study, every group was considered an independent data set. If the HRs with 95% CIs for outcomes were not shown directly in the article, the survival data were extracted from Kaplan-Meier plots by Engauge Digitizer 4.1 software. Then, the HRs and 95% CIs were calculated using the Excel program file provided by Tierney et al. [20].

### Quality assessment and publication bias

The quality of the retrieved papers was evaluated with the Newcastle-Ottawa Scale (NOS) criteria. Points 5–9 are considered high quality, and points 1–4 are considered low quality [21]. Two reviewers (HJ and XJG) independently evaluated the quality of the selected papers. If necessary, disagreements were resolved by a third reviewer (ZHZ). Publication bias was examined visually using funnel plots and statistically assessed using Egger’s test [22]. A p-value < 0.05 was considered statistically significant. If necessary, the trim and fill method was used to assess the influence of potential publication bias on the pooled results [23].

### Statistical analysis

The meta-analysis was performed in STATA software (Version 15.1). The HR with 95% CI was chosen to evaluate outcomes of survival (OS, CSS, PFS/TTP and DFS/RFS/TFR) according to Parmar et al. [24]. HR > 1 implies poor survival in the CTC-positivity group, and *p* < 0.05 was considered statistically significant. Heterogeneity was assessed using the Q test and I^2^ statistics. I^2^ < 30% was considered nonimportant, 30–60% was moderate, and >60% was substantial. The random-effect model was used for analysis [25]. Then, according to the heterogeneity of the data, subgroup analyses were conducted based on the numbers of patients, methods, positivity rates, and follow-up time. The stability of the pooled results was assessed by one-way sensitivity analysis. The article complied with the Quality of Reporting of Meta-Analyses (QUORUM) and the Preferred Reporting Items for Systematic Reviews and Meta-Analyses (PRISMA) statement [26].

## Results

### Baseline study characteristics

**Fig 1:**
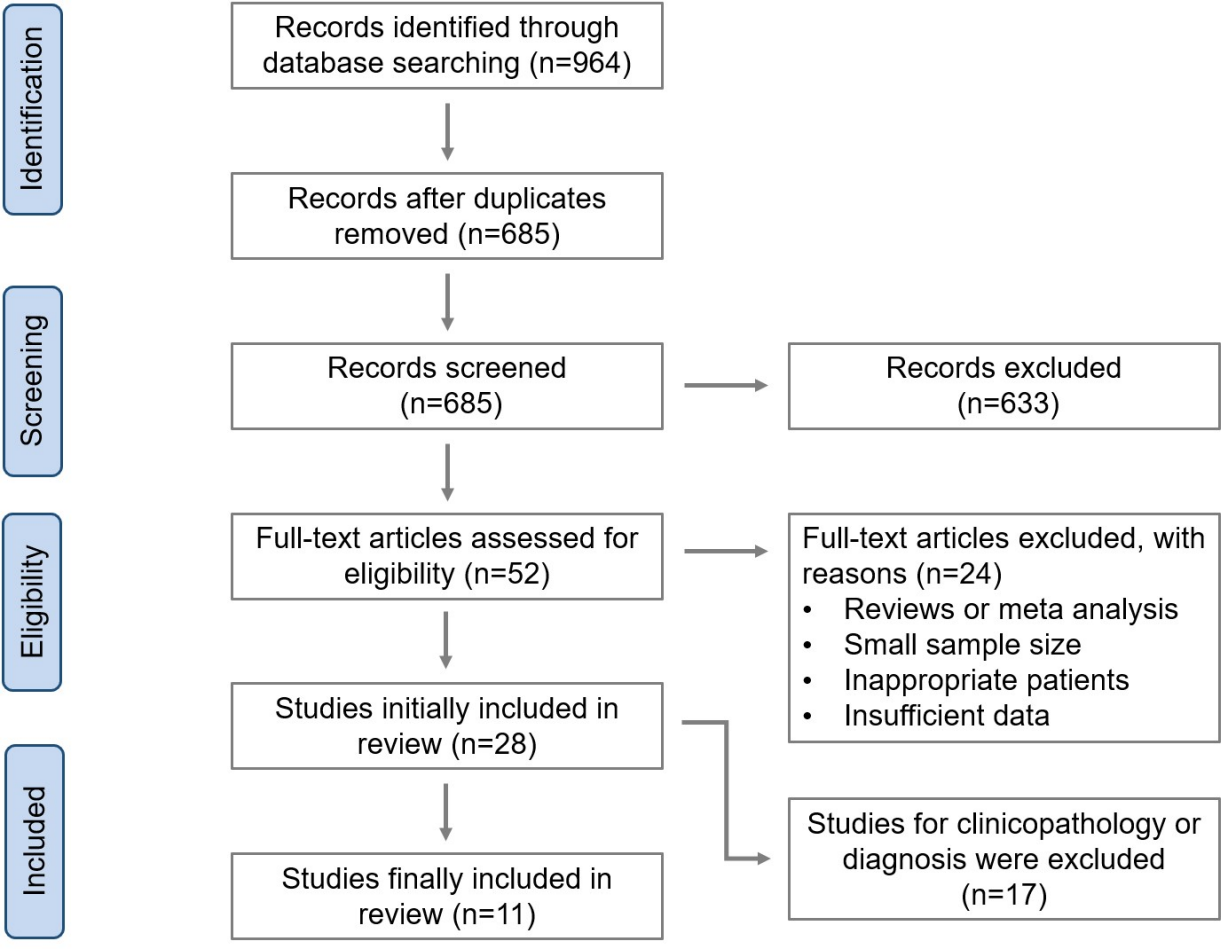
Flow chart for selecting eligible studies.

Fig 1 shows the flow diagram for study selection. A total of 964 publications relevant to the study topic were selected for preliminary screening. After excluding duplicates, 685 records remained. Following the review of titles and abstracts, 633 non-relevant papers were filtered out. The remaining 52 full manuscripts were subjected to detailed reading, of which 24 papers were further excluded due to inappropriate article type, small sample size, inappropriate patient selection, or insufficient data, and 17 studies on the relationship between CTCs and the diagnosis of BC were also excluded. Finally, the meta-analysis contained only 11 eligible studies on the relationship between CTCs and the prognosis of BC, for a total of 1062 included patients [27–37].

Table 1 shows the basic characteristics of the included articles. These studies were published between 2011 and 2019, of which four studies were conducted in patients with BC, one study was performed in patients with advanced BC, and the other six studies included only patients with NMIBC. The median number of patients was 88 (range: 44–188). The detection methods included the CellSearch system and CELLection Dynabeads. The median positive rate was 21% (range: 17.8%–44.4%), and the median follow-up time was 39 months (range 24–108). According to the quality assessment by the Newcastle-Ottawa Scale (NOS), 10 of the 11 eligible articles were considered to be high quality (Table 2).

**Table 1:** Baseline characteristics of the included studies.

Author/Year	Patients	No. of patients	Detection methods	Positive rate (%)	Follow-up (months)	Outcomes	Multi variant
Abrahamsson, J. 2017 [27]	BC	75	CellSearch system	18	42	CSS, PFS	Yes
Busetto, G. M. 2017 [28]	NMIBC	101	CellSearch system	19.8	28	TFR, TTP	Yes
Busetto, G. M. 2 2017 [28]	NMIBC	54	CELLection Dynabeads	44.4	28	TFR, TTP	Yes
Gazzaniga, P. 2014 [29]	NIMBC	102	CellSearch system	20	36	TFR, TTP	Yes
Gazzaniga, P. 2012 [30]	NMIBC	44	CellSearch system	18	24	TFR	No
Gradilone, A. 2010 [31]	NMIBC	54	CELLection Dynabeads	44	24	DFS	Yes
Nicolazzo, C. 2017 [32]	NMIBC	54	CELLection Dynabeads	44	108	CSS, DFS	No
Nicolazzo, C. 2019 [33]	NMIBC	102	CellSearch system	20	90	CSS, OS, TFR, TTP	Yes
Rink, M. 2012 [34]	BC	100	CellSearch system	23	45	CSS, OS, RFS	Yes
Rink, M. 2011 [35]	Advanced BC	53	CellSearch system	36.4	30	OS, PFS	No
Soave, A. 2017 (BJUI) [36]	BC	188	CellSearch system	22.3	46	CSS, RFS	Yes
Soave, A. 2017 (IJC) [37]	BC	135	CellSearch system	17.8	84	CSS, OS, RFS	Yes

**Table 2:** Quality assessment of the included studies according to the Newcastle-Ottawa scale.

Author/Year	①	②	③	④	⑤	⑥	⑦	⑧	Total points
Abrahamsson, J. 2017 [27]	0	1	1	1	2	1	1	0	7
Busetto, G. M. 2017 [28]	1	1	1	1	2	1	0	1	8
Busetto, G. M. 2 2017 [28]	1	1	1	1	2	1	0	1	8
Gazzaniga, P. 2014 [29]	1	1	1	1	2	1	1	0	8
Gazzaniga, P. 2012 [30]	0	1	1	1	0	1	0	1	5
Gradilone, A. 2010 [31]	1	1	1	1	2	1	0	1	8
Nicolazzo, C. 2017 [32]	0	1	1	1	0	1	1	1	6
Nicolazzo, C. 2019 [33]	1	1	1	1	2	1	1	1	9
Rink, M. 2012 [34]	0	1	1	1	2	1	1	1	8
Rink, M. 2011 [35]	0	1	1	1	0	1	0	0	4
Soave, A. 2017 (BJUI) [36]	0	1	1	1	2	1	1	1	8
Soave, A. 2017 (IJC) [37]	0	1	1	1	2	1	1	0	7

Notes: **①**Representativeness of the exposed cohort (1 point); **②**Representativeness of the non-exposed cohort (1 point); **③**Ascertainment of exposure (1 point); **④**Demonstration that outcome of interest was not present at the start of the study (1 point); **⑤**Comparability of cohorts on the basis of the design or analysis (2 points); **⑥**Assessment of outcome (1 point); **⑦**Was follow up long enough for outcomes to occur (1 point); **⑧**Adequacy of follow up of cohorts (1 point).

### Impact of CTC positivity on prognostic effects related to OS and CSS

**Fig 2:**
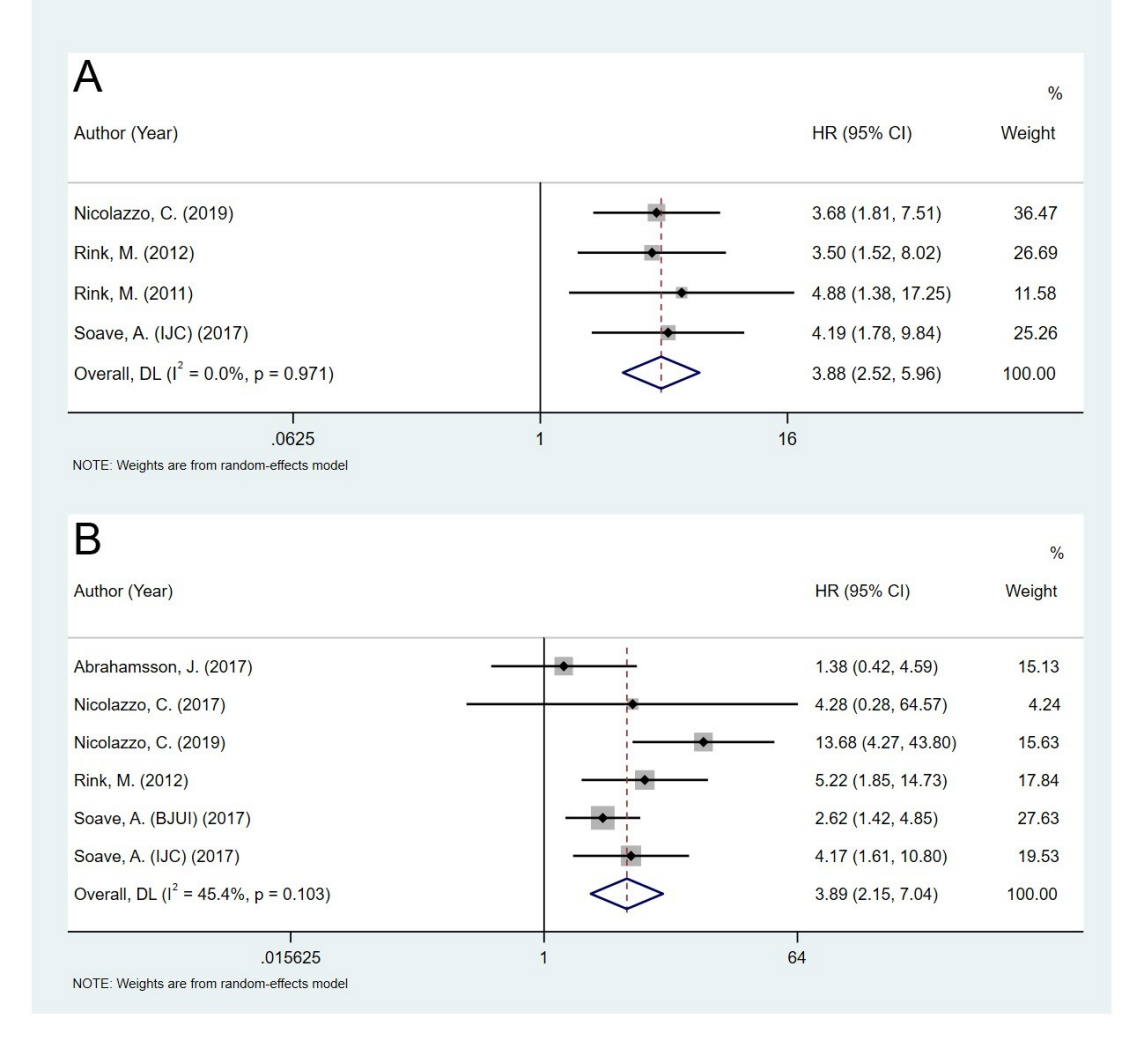
Pooled HRs for overall survival and cancer-free survival of patients in the included studies. A: overall survival. B: cancer-free survival.

The pooled hazard ratio (HR) for OS was analyzed in four studies that included 390 patients. The results demonstrated that the presence of CTCs was highly correlated with the prognosis of poor OS (HR = 3.88, 95% CI 2.52–5.96, *p* < 0.001, Fig 2A), and no significant heterogeneity was observed between the studies (I^2^ = 0.0%, *p* = 0.971). Six studies that included 654 patients were analyzed for CSS. The results showed that the presence of CTCs was highly correlated with poor CSS (HR = 3.89, 95% CI 2.15–7.04, *p* < 0.001, Fig 2B), with moderate heterogeneity (I^2^ = 45.4%, *p* = 0.103). These findings demonstrated that the risk of both overall mortality and disease-associated mortality increased with the CTC positivity rate of patients with BC.

### Impact of CTC positivity on prognostic effects related to PFS/TTP and DFS/RFS/TFR

**Fig 3:**
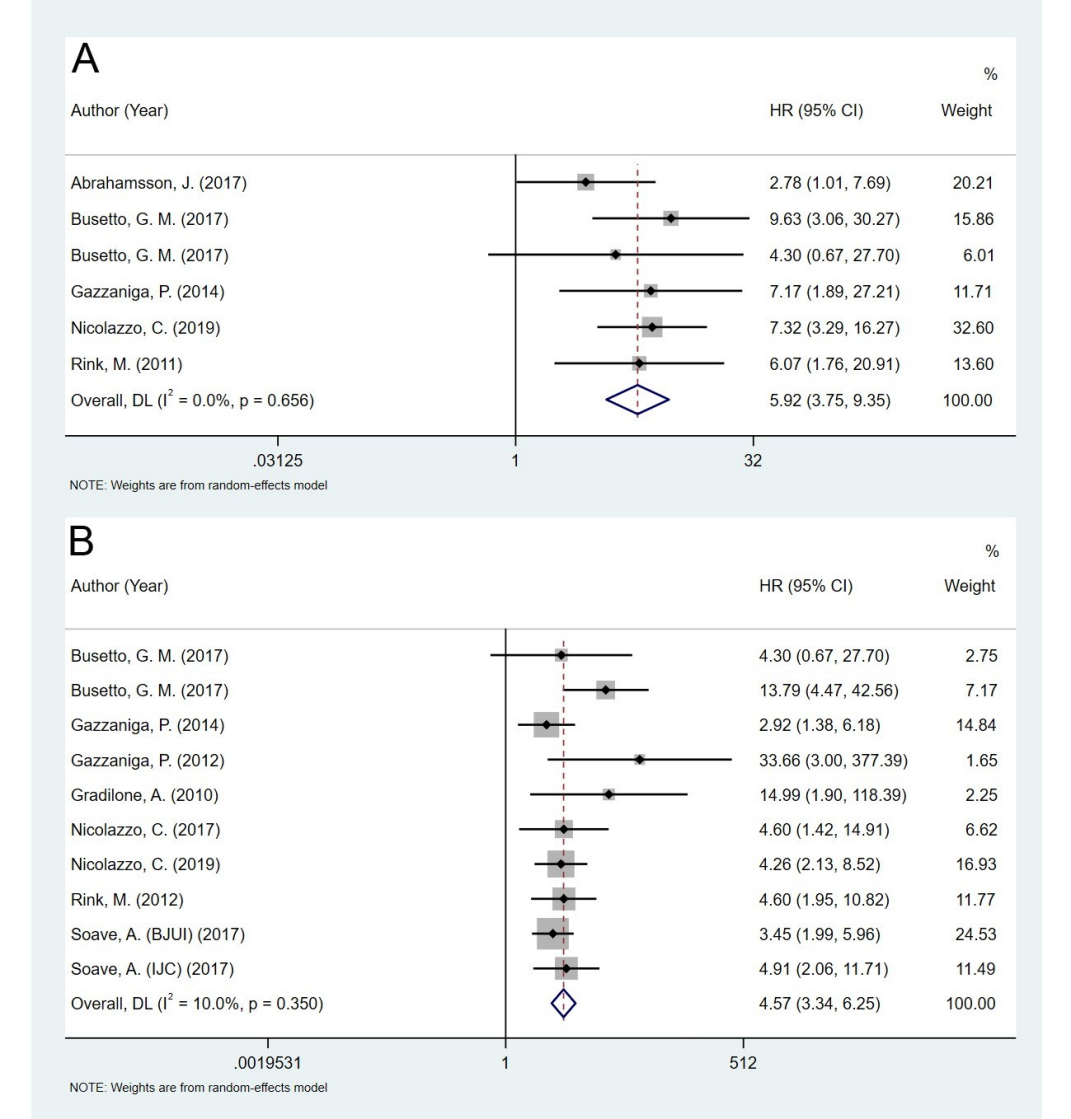
Pooled HRs for progression-free survival/time to progression and disease-free survival/recurrence-free survival/time to first recurrence of patients in the included studies. A: progression-free survival/time to progression. B: disease-free survival/recurrence-free survival/time to first recurrence.

The HRs for PFS/TTP were available in six studies representing 487 patients. The pooled HR revealed that the presence of CTCs predicted worse outcome for patients with BC (HR = 5.92, 95% CI 3.75–9.35, *p* < 0.001, Fig 3A), and no significant heterogeneity was observed between the studies (I^2^ = 0.0%, *p* = 0.656). The pooled HR for DFS/RFS/TFR was analyzed in 10 studies that included 934 patients. The results showed a high risk of tumor recurrence in the CTC-positive group (HR = 4.57, 95% CI 3.34–6.25, *p* < 0.001, Fig 3B), with mild heterogeneity (I^2^ = 10.0%, *p* = 0.350). These findings demonstrated that the presence of CTCs predicted an increased risk of both disease progression and recurrence for patients with BC.

### Subgroup analyses

In the subgroup analyses based on the number of patients (divided into ≥ median or not), significant prognostic effects (OS, PFS/TTP, and DFS/RFS/TFR) of CTCs were observed in these groups (Table 3). However, in the analyses of studies with patient number < median cases, no significant differences in death risk (CSS) were found between the CTC-positive and CTC-negative groups. For subgroup analyses based on the detection method, the results showed that CTC positivity reliably predicted worse prognosis (OS, CSS, PFS/TTP, and DFS/RFS/TFR) with detection using the CellSearch system. Moreover, regardless of whether the CellSearch system or CELLection Dynabeads was used for tumor detection, the presence of CTCs in the bloodstream could predict malignant progression (DFS/RFS/TFR).

**Table 3:** Subgroup analyses of HRs based on different numbers of patients and detection methods.

		Patients’ no. ≥ median		Methods	
		Yes	No	CellSearch system	CELLection Dynabeads
OS	N	3	1	4	0
	HR (95% CI)	3.76 (2.38–5.94)	4.88 (1.38–17.25)	3.88 (2.52–5.96)	-
	I^2^ (%)	0.0	-	0.0	-
	*p*	<0.001	0.014	<0.001	-
CSS	N	4	2	5	1
	HR (95% CI)	4.71 (2.42–9.18)	1.66 (0.56–4.95)	3.90 (2.04–7.46)	4.28 (0.28–65.00)
	I^2^ (%)	52.9	0.0	56.2	-
	*p*	<0.001	0.366	<0.001	0.295
PFS/TTP	N	3	3	5	1
	HR (95% CI)	7.84 (4.35–14.11)	3.88 (1.88–7.99)	6.04 (3.78–9.68)	4.30 (0.67–27.65)
	I^2^ (%)	0.0	0.0	0.0	-
	*p*	<0.001	<0.001	<0.001	0.124
DFS/RFS/TFR	N	6	4	7	3
	HR (95% CI)	3.83 (2.80–5.24)	9.67 (4.84–20.53)	3.97 (2.91–5.42)	8.85 (4.15–18.86)
	I^2^ (%)	0.0	3.4	0.0	1.7
	*p*	<0.001	<0.001	<0.001	<0.001

Notes: HR, hazard ratio; I^2^, I-squared; *p*, the *p*-value for HR estimates; The median patients’ number was 88; -, not available.

The predictive role of CTCs in disease survival and progression (OS, CSS, PFS/TTP, and DFS/RFS/TFR) was also demonstrated in both groups of CTC positivity (Table 4). In the subgroup analyses of follow-up time, the detection of CTCs revealed a predictive value for both disease survival and progression (OS, PFS/TTP, and DFS/RFS/TFR) for patients with BC. We did not perform subgroup analyses for NMIBC and MIBC due to a lack of information on patients with MIBC. Nevertheless, the prognostic use of CTCs in NMIBC was still confirmed (S1 Table).

**Table 4:** Subgroup analyses of HRs based on different positivity rates and follow-up times.

		Positivity rates ≥ median		Follow-up time ≥ 36 months	
		Yes	No	Yes	No
OS	N	2	2	3	1
	HR (95% CI)	3.87 (1.93–7.75)	3.88 (2.25–6.71)	3.76 (2.38–5.94)	4.88 (1.38–17.25)
	I^2^ (%)	0.0	0.0	0.0	-
	*p*	<0.001	<0.001	<0.001	0.014
CSS	N	3	3	6	0
	HR (95% CI)	3.17 (1.89–5.32)	4.31 (1.28–14.46)	3.60 (2.42–5.37)	-
	I^2^ (%)	0.0	72.5	45.4	-
	*p*	<0.001	0.018	<0.001	-
PFS/TTP	N	2	4	3	3
	HR (95% CI)	5.46 (1.95–15.30)	6.04 (3.63–10.05)	5.39 (3.05–9.50)	7.04 (3.27–15.14)
	I^2^ (%)	0.0	5.3	15.8	0.0
	*p*	0.001	<0.001	<0.001	<0.001
DFS/RFS/TFR	N	5	5	6	4
	HR (95% CI)	4.73 (3.19–7.02)	4.17 (2.73–6.34)	3.87 (2.84–5.26)	12.34 (5.43–28.07)
	I^2^ (%)	32.7	0.0	0.0	0.0
	*p*	<0.001	<0.001	<0.001	<0.001

Notes: HR, hazard ratio; I^2^, I-squared; *p*, the *p*-value for HR estimates; The median positive rate was 21%; -, not available.

### Sensitivity analysis and publication bias

Sensitivity analysis was performed by deleting one study at a time from the overall pooled analysis. The results showed that our pooled results were relatively stable for OS, CSS, PFS/TTP, and DFS/RFS/TFR (S1 Fig). Moreover, funnel plots and Egger’s test were performed to evaluate the publication bias in the DFS/RFS/TFR group. The results of funnel plots showed that the distribution of dots was asymmetric (S2 Fig), thus implying a possible publication bias among the included studies, which was also confirmed by Egger’s test (*p* < 0.05). The trim and fill method was then applied to adjust for publication bias. After applying this method, the pooled HR was still significant (HR 3.80, 95% CI 2.62–5.52, *p* < 0.001), indicating that our results were stable.

## Discussion

Bladder cancer is one of the most common diseases of the urinary system. With advanced in tumor stage, the 5-year survival rate of patients was found to decrease sharply, with a survival rate of as low as 15% in patients with stage 4 tumors [6]. Therefore, early detection and early intervention of tumor metastasis could improve the survival rate of patients. The standard detection methods for follow-up include cystoscopy, urine cytology, and contrast-enhanced computed tomography scan. However, cystoscopy and urine cytology lack effectiveness to capture metastatic tumors, while an enhanced CT scan requires a certain tumor volume to diagnose tumors [38, 39]. In recent years, CTCs have shown potential in the early screening of cancers, predicting outcomes, and monitoring the treatment responses of various tumors. Specifically, CTCs were found to predict distant metastasis of tumors and provide abundant molecular information on tumors *in situ* [40, 41].

Numerous cohort studies in patients with BC have shown that the detection of CTCs provides meaningful information on cancer diagnosis and prognosis. In 2011, Pavlos et al. conducted a systematic review and meta-analysis that demonstrated the diagnostic value of CTC detection in BC and UC. In 2017, Zheng et al. published an updated meta-analysis to evaluate the diagnostic and prognostic role of CTCs in BC and upper tract urothelial carcinoma. However, previous studies have not adequately focused on BC nor have they performed subgroup analysis for the prognostic role of CTCs. In the present meta-analysis, we demonstrated that the presence of CTCs in the bloodstream indicated poor prognoses for patients with BC (Figs 2 and 3). CTCs are better prognostic markers for tumor recurrence and progression (PFS/TTP and DFS/RFS/TFR) than for survival time (OS and CSS). These study findings may help provide a rationale for the use of CTCs for the prognosis and therapy of BC.

When the studies were divided into two groups according to the number of patients, the pooled results of the group with a lower number of patients (n < median) failed to reach statistical significance in terms of CSS; thus, reliable results are dependent on studies with large samples (Table 3). Currently, the CellSearch system [42] and CELLection Dynabeads [43, 44] are the most widely used methods to detect CTCs in peripheral blood. For detection using the CellSearch system, the CTC-positivity status was significantly associated with poorer prognoses (OS, CSS, PFS/TTP, and DFS/RFS/TFR) compared to the CTC-negativity status, thus implying that the CellSearch system is an effective method to detect CTCs. However, for detection using CELLection Dynabeads, a predictive role of CTCs for disease recurrence (DFS/RFS/TFR) was observed, indicating that the CellSearch system might be a more accurate tool for assessing CTCs than CELLection Dynabeads. Moreover, the results of subgroup analyses based on follow-up time showed an increase in 95% CI in the group with <36 months of follow-up compared to that in the group with ≥36 months of follow-up (Table 4); this finding suggested that adequate follow-up time was an essential factor for increasing the reliability of the results. Although the 5-year survival rate of patients with NMIBC was quite satisfactory, they tended to relapse and progress to MIBC. Our results showed that the presence of CTCs in patients with NMIBC was a biomarker for prognosis (S1 Table), implying that the detection of CTCs might help to diagnose the recurrence and metastasis of tumors early, contributing to the timely treatment of patients with NMIBC.

Moderate heterogeneity between the included studies was observed in the CSS group (I^2^ = 45.4%). After subgroup analyses based on the number of patients, positivity rate, and subtype of BC, the heterogeneity partly decreased, suggesting that these factors might be the source of heterogeneity. An adequate number of studies are required in a meta-regression analysis; for example at least 10 observations are needed for each covariate model. Because the number of studies included in this meta-analysis was relatively low, we did not perform a meta-regression analysis. However, by conducting sensitivity analyses, we confirmed the stability and reliability of the pooled results. Although the results of funnel plots and Egger’s test showed that publication bias existed in the DFS/RFS/TFR group, the trim-and-fill method confirmed the stability of the pooled result.

The present meta-analysis had some limitations. First, for the HR and 95% CI values reported in our meta-analysis, some data were not original data from the included studies and were estimated from Kaplan-Meier plots. Second, only a few studies have used CELLection Dynabeads to identify CTCs; hence, we could not thoroughly compare the diagnostic ability of the CellSearch system and CELLection Dynabeads to detect CTCs. Third, several studies did not provide a definite diagnosis of the disease or differentiated between subtypes of BC; hence, we could not obtain information on the prognostic effects of CTCs in patients with NMIBC vs MIBC. Fourth, the numbers of the included studies in the OS, CSS, and PFS/TTP analyses were less than 10; therefore, we did not conduct an assessment of publication bias for these groups. Finally, PFS was defined as the time from the start of randomization to the tumor’s progression or death (due to any reason), while TTP was defined as the time from the start of randomization to tumor progression. For a limited number of included studies, we combined PFS with TTP to form an outcome; the outcome DFS/RFS/TFR was also combined for the same reason. Therefore, higher-quality cohort studies are required to investigate the prognostic role of CTCs in patients with BC.

## Conclusion

Our meta-analysis results strongly indicated that the presence of CTCs in the bloodstream was a clinically promising prognostic biomarker of disease survival and progression for patients with BC. In the future, higher-quality cohort studies are needed to confirm our conclusions.

## Supporting information

S1 Fig Sensitivity analysis in the included studies. A: OS B: CSS C: PFS/TTP D: DFS/RFS/TFR.
(TIF)

S2 Fig Funnel plots for the publication bias in the DFS/RFS/TFR group. (TIF)

S1 Table Impact of CTC-positivity on the prognostic effects in NMIBC. (DOCX)

S1 Text Full search strategy. (DOCX)

S1 File A protocol for this meta-analysis. (PDF)

S2 File PRISMA checklist. (DOC)
