# Supplementary figures and images for: Prognostic value of circulating tumor cells in patients with bladder cancer: A meta-analysis
Source: PLoS One. 2021 Jul 9;16(7):e0254433. doi: 10.1371/journal.pone.0254433 (PMC8270423; doi:10.1371/journal.pone.0254433)

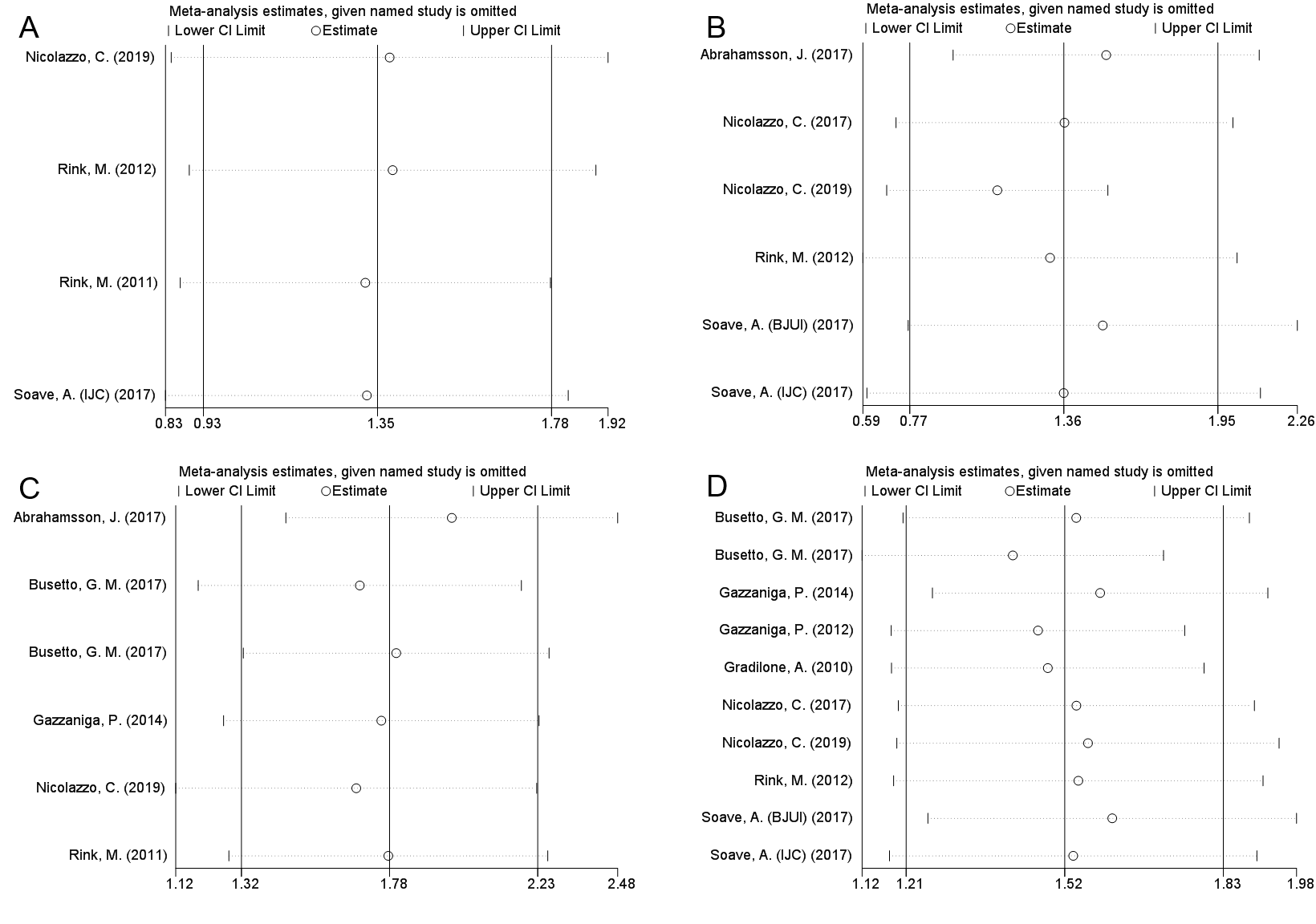

Supplement: S1 Fig — A: OS B: CSS C: PFS/TTP D: DFS/RFS/TFR. (TIF) [file pone.0254433.s001.tif]

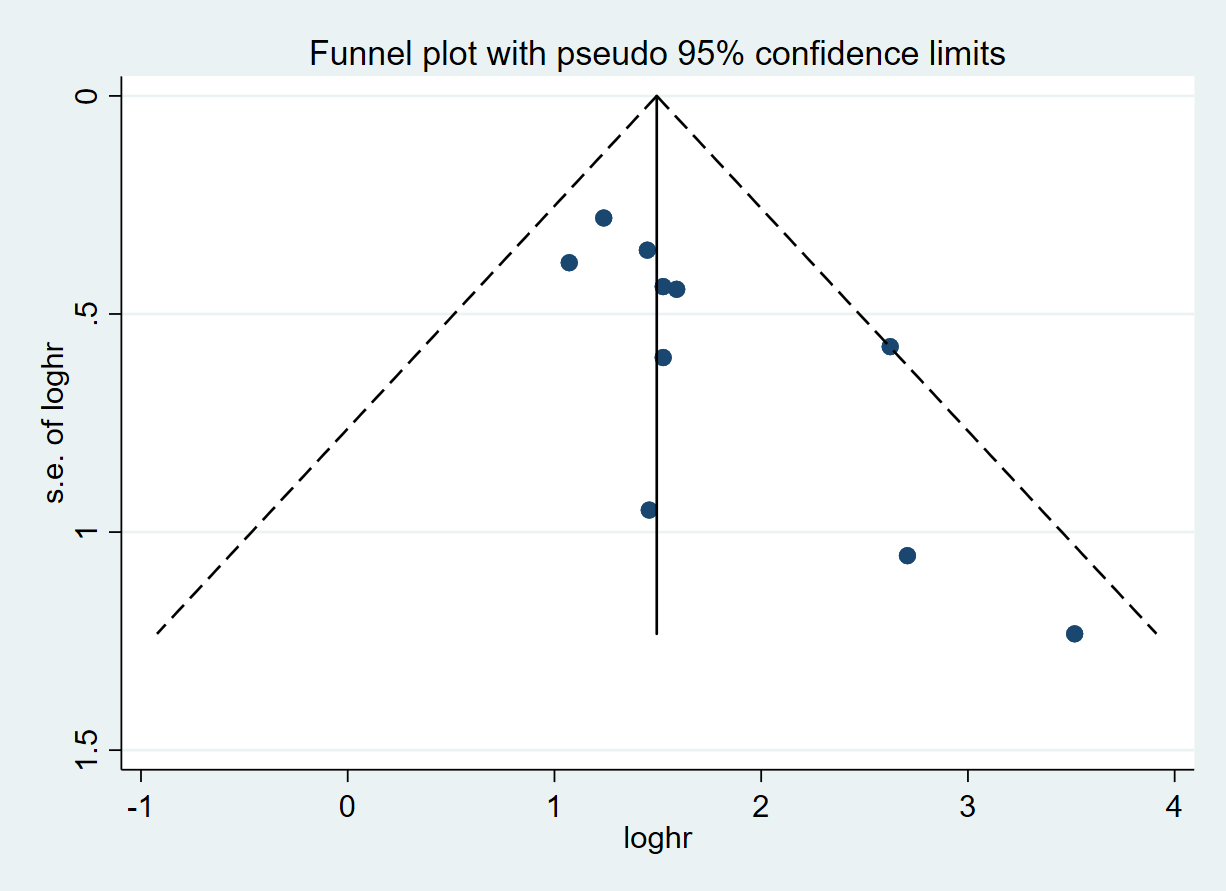

Supplement: S2 Fig — (TIF) [file pone.0254433.s002.tif]
