# Supplementary material for: Prognostic value of circulating tumor cells in patients with bladder cancer: A meta-analysis
Source: PLoS One. 2021 Jul 9;16(7):e0254433. doi: 10.1371/journal.pone.0254433 (PMC8270423; doi:10.1371/journal.pone.0254433)
Supplement: S1 Table — (DOCX) [file pone.0254433.s003.docx]

**S1 Table. Impact of CTC-positivity on the prognostic effects in NMIBC subgroup.**

|  |  | OS | | CSS | | PFS/TTP | | DFS/RFS/TFR |
| --- | --- | --- | --- | --- | --- | --- | --- | --- |
| NMIBC | N | 1 | 2 | | 4 | | 7 | |
|  | HR (95% CI) | 3.68 (1.81-7.50) | 11.43 (3.92-33.32) | | 7.42 (4.24-13.00) | | 5.58 (3.25-9.60) | |
|  | I^2^ (%) | - | 0.0 | | 0.0 | | 31.9 | |
|  | *p* | <0.001 | <0.001 | | <0.001 | | <0.001 | |
