# Supplementary material for: Prognostic value of circulating tumor cells in patients with bladder cancer: A meta-analysis
Source: PLoS One. 2021 Jul 9;16(7):e0254433. doi: 10.1371/journal.pone.0254433 (PMC8270423; doi:10.1371/journal.pone.0254433)
Supplement: S1 Text — (DOCX) [file pone.0254433.s004.docx]

# **S1 Text. Full search strategies.**

The detailed search strategy used in PubMed was: (("neoplastic cells, circulating"[MeSH Terms] OR circulating tumor cells [Text Word] OR CTCs [Text Word]) AND ("Urinary Bladder Neoplasms"[Mesh] OR bladder cancer [Text Word] OR bladder transitional cell carcinoma [Text Word] OR urothelial carcinoma of the bladder[Text Word] OR urothelial cancer[Text Word])) AND (English[Language])

The search date: 10 March 2021

The number of the results: 151

The detailed search strategy used in Web of Science was:

#1 (TS=(neoplastic cells, circulating OR circulating tumor cells OR CTCs) ) AND Language: (English)

#2 (TS=(Urinary Bladder Neoplasms OR bladder cancer OR bladder transitional cell carcinoma OR urothelial carcinoma of the bladder OR urothelial cancer) ) AND Language: (English)

#3 #1 AND #2

The search date: 10 March 2021

The number of the results: 525

The detailed search strategy used in Embase was:

#1 'neoplastic cells, circulating'/exp OR 'circulating tumor cells':ab,ti OR 'ctcs':ab,ti

#2 'urinary bladder neoplasms'/exp OR 'bladder cancer':ab,ti OR 'bladder transitional cell carcinoma':ab,ti OR 'urothelial carcinoma of the bladder':ab,ti OR 'urothelial cancer':ab,ti

#3 english:la

#4 #1 AND #2 AND #3

The search date: 10 March 2021

The number of the results: 288
