# Supplementary material for: Prognostic value of circulating tumor cells in patients with bladder cancer: A meta-analysis
Source: PLoS One. 2021 Jul 9;16(7):e0254433. doi: 10.1371/journal.pone.0254433 (PMC8270423; doi:10.1371/journal.pone.0254433)
Supplement: S1 File — (PDF) [file pone.0254433.s005.pdf]

Systematic review

This record cannot be edited because it is being assessed by the editorial team

1. * Review title.

Give the title of the review in English

The prognostic value of circulating tumor cells in bladder cancer patients: a meta-analysis

2. Original language title.

For reviews in languages other than English, give the title in the original language. This will be displayed with the English language title.

3. * Anticipated or actual start date.

Give the date the systematic review started or is expected to start.

16/11/2020

4. * Anticipated completion date.

Give the date by which the review is expected to be completed.

01/05/2021

5. * Stage of review at time of this submission.

Tick the boxes to show which review tasks have been started and which have been completed.
Update this field each time any amendments are made to a published record.

Reviews that have started data extraction (at the time of initial submission) are not eligible for inclusion in PROSPERO.
If there is later evidence that incorrect status and/or completion date has been supplied, the published PROSPERO record will be marked as retracted.

This field uses answers to initial screening questions. It cannot be edited until after registration.

The review has not yet started: No

Review stage	Started	Completed
Preliminary searches	Yes	No
Piloting of the study selection process	Yes	No
Formal screening of search results against eligibility criteria	No	No
Data extraction	No	No
Risk of bias (quality) assessment	No	No
Data analysis	No	No

Provide any other relevant information about the stage of the review here.

6. * Named contact.

The named contact is the guarantor for the accuracy of the information in the register record. This may be any member of the review team.

Hui Jiang

Email salutation (e.g. "Dr Smith" or "Joanne") for correspondence:
Miss Jiang

7. * Named contact email.

Give the electronic email address of the named contact.

jianghui@swmu.edu.cn

8. Named contact address

PLEASE NOTE this information will be published in the PROSPERO record so please do not enter private information, i.e. personal home address

Give the full institutional/organisational postal address for the named contact.

Department of Laboratory Medicine, the Affiliated Hospital of Southwest Medical University, 25 Taiping street, Luzhou 646000, P.R. China

9. Named contact phone number.

Give the telephone number for the named contact, including international dialling code.

13667643213

10. * Organisational affiliation of the review.

Full title of the organisational affiliations for this review and website address if available. This field may be completed as 'None' if the review is not affiliated to any organisation.

Department of Laboratory Medicine, Affiliated Hospital of Southwest Medical University

Organisation web address:

11. * Review team members and their organisational affiliations.

Give the personal details and the organisational affiliations of each member of the review team. Affiliation refers to groups or organisations to which review team members belong.
NOTE: email and country now MUST be entered for each person, unless you are amending a published record.

Miss Hui Jiang. Department of Laboratory Medicine, The Affiliated Hospital of Southwest Medical University, Luzhou, China

Miss Xiujuan Gu. Department of Laboratory Medicine, The Affiliated Hospital of Southwest Medical University

Mr Zhihua Zuo. Department of Laboratory Medicine, The Affiliated Hospital of Southwest Medical University, Luzhou, China

Dr Gang Tian. Department of Laboratory Medicine, The Affiliated Hospital of Southwest Medical University, Luzhou, China

Professor Jinbo Liu. Department of Laboratory Medicine, The Affiliated Hospital of Southwest Medical University, Luzhou, China

12. * Funding sources/sponsors.

Details of the individuals, organizations, groups, companies or other legal entities who have funded or sponsored the review.

Sichuan Science and Technology Program

Grant number(s)

State the funder, grant or award number and the date of award

2019YFS0332, 2019YFH0010, 2019YFS0038, 21ZDYF2160 and 21GJHZ0317

13. * Conflicts of interest.

List actual or perceived conflicts of interest (financial or academic).

None

14. Collaborators.

Give the name and affiliation of any individuals or organisations who are working on the review but who are not listed as review team members. **NOTE: email and country must be completed for each person, unless you are amending a published record.**

15. * Review question.

State the review question(s) clearly and precisely. It may be appropriate to break very broad questions down into a series of related more specific questions. Questions may be framed or refined using PI(E)COS or similar where relevant.

How does circulating tumor cells status impact on survival of bladder cancer patients?

16. * Searches.

State the sources that will be searched (e.g. Medline). Give the search dates, and any restrictions (e.g. language or publication date). Do NOT enter the full search strategy (it may be provided as a link or attachment below.)

Sources include PubMed, Embase and Web of Science.
Search date was in 28 November 2020.
The language is limited to English.

17. URL to search strategy.

Upload a file with your search strategy, or an example of a search strategy for a specific database, (including the keywords) in pdf or word format. In doing so you are consenting to the file being made publicly accessible.

Or provide a URL or link to the strategy. Do NOT provide links to your search **results**.

Do not make this file publicly available until the review is complete

18. * Condition or domain being studied.

Give a short description of the disease, condition or healthcare domain being studied in your systematic review.

Bladder cancer (BC) ranks as the tenth most frequently diagnosed cancer worldwide. In 2018, there were approximately 550 000 new cases and 200 000 deaths of BC, 80% of which occurred in men. According to the different treatment modes and prognosis, BC is subdivided into two types (non-muscle-invasive bladder cancer (NMIBC) and muscle-invasive bladder cancer (MIBC)). NMIBC tends to relapse, with which patients need a long-term monitoring, while aggressive MIBC is prone to metastasis, and is associated with a high rate of mortality. Nevertheless, BC's overall therapeutic effects are not satisfactory, and the five-year survival rate is pretty low. Therefore, searching for a risk assessment tool for prognosis can be beneficial for treating patients with BC.

19. * Participants/population.

Specify the participants or populations being studied in the review. The preferred format includes details of both inclusion and exclusion criteria.

The inclusion criteria were: (1) studies investigating the association between CTCs' status and prognosis of BC patients; (2) studies providing HR with 95% CI or Kaplan-Meier curves; (3) studies enrolling more than 20 patients.

The exclusion criteria were: (1) review, letter, conference abstract or case report; (2) samples were not collected from peripheral blood.

20. * Intervention(s), exposure(s).

Give full and clear descriptions or definitions of the interventions or the exposures to be reviewed. The preferred format includes details of both inclusion and exclusion criteria.

Circulating tumor cells positive in serum was the main exposure.

21. * Comparator(s)/control.

Where relevant, give details of the alternatives against which the intervention/exposure will be compared (e.g. another intervention or a non-exposed control group). The preferred format includes details of both inclusion and exclusion criteria.

Circulating tumor cells negative group was the control.

22. * Types of study to be included.

Give details of the study designs (e.g. RCT) that are eligible for inclusion in the review. The preferred format includes both inclusion and exclusion criteria. If there are no restrictions on the types of study, this should be stated.

Prospective study will be included.

23. Context.

Give summary details of the setting or other relevant characteristics, which help define the inclusion or exclusion criteria.

24. * Main outcome(s).

Give the pre-specified main (most important) outcomes of the review, including details of how the outcome is defined and measured and when these measurement are made, if these are part of the review inclusion criteria.

overall survival (OS) and cancer-specific survival (CSS)

* Measures of effect
Hazard ratios

25. * Additional outcome(s).

List the pre-specified additional outcomes of the review, with a similar level of detail to that required for main outcomes. Where there are no additional outcomes please state ‘None’ or ‘Not applicable’ as appropriate to the review

progression-free survival (PFS)/time to progression (TTP) and disease-free survival (DFS)/recurrence-free survival (RFS)/time to first recurrence (TFR)

* Measures of effect
Hazard ratios

26. * Data extraction (selection and coding).

Describe how studies will be selected for inclusion. State what data will be extracted or obtained. State how this will be done and recorded.

Two people will independently extract data. If necessary, disagreements will be resolved by a third people.
The following data will be extracted: name of the first author, publication year, diagnosis, number of patients, detection method, positive rate, follow-up months and outcomes.

27. * Risk of bias (quality) assessment.

State which characteristics of the studies will be assessed and/or any formal risk of bias/quality assessment tools that will be used.

Newcastle.Ottawa scale (NOS) will be used for quality assessment.
The assessed characteristics will include:
1. Representativeness of the exposed cohort; 2. Representativeness of the non-exposed cohort; 3. Ascertainment of exposure; 4. Demonstration that outcome of interest was not present at the start of study; 5. Comparability of cohorts on the basis of the design or analysis; 6. Assessment of outcome; 7. Was follow up long enough for outcomes to occur; 8. Adequacy of follow up of cohorts.
Two reviewers will be involved in the quality assessment. If necessary,
disagreements will be resolved by a third reviewer.

28. * Strategy for data synthesis.

Describe the methods you plan to use to synthesise data. This **must not be generic text** but should be **specific to your review** and describe how the proposed approach will be applied to your data.
If meta-analysis is planned, describe the models to be used, methods to explore statistical heterogeneity, and software package to be used.

Hazard ratio (HR) for both fixed and random effects models (weighting by inverse of variance) will be used. Between-study heterogeneity will be assessed using the Q test and I^2 statistics. According to the Cochrane handbook, the I^2 will be considered non-important (<30%), moderate (30%-60%) and substantial (>60%). Results will be assessed using forest plots and presented as HRs for the outcomes. An influence analysis will be performed to ascertain the results of the meta-
analysis by excluding each of the individual studies. Publication bias will be assessed by a funnel plot for meta-analysis and quantified by the Egger method. Statistical analysis will be conducted using STATA software.

29. * Analysis of subgroups or subsets.

State any planned investigation of 'subgroups'. Be clear and specific about which type of study or participant will be included in each group or covariate investigated. State the planned analytic approach.

We will consider subgroups such as patients' number, detection method, positive rate and follow-up months.

30. * Type and method of review.

Select the type of review, review method and health area from the lists below.

Type of review

Cost effectiveness	No
Diagnostic	No
Epidemiologic	No
Individual patient data (IPD) meta-analysis	No
Intervention	No
Meta-analysis	Yes
Methodology	No
Narrative synthesis	No
Network meta-analysis	No
Pre-clinical	No

Prevention	No
Prognostic	Yes
Prospective meta-analysis (PMA)	Yes
Review of reviews	No
Service delivery	No
Synthesis of qualitative studies	No
Systematic review	Yes
Other	No

Health area of the review

Alcohol/substance misuse/abuse	No
Blood and immune system	No
Cancer	Yes
Cardiovascular	No
Care of the elderly	No
Child health	No
Complementary therapies	No
COVID-19	No
Crime and justice	No
Dental	No
Digestive system	No
Ear, nose and throat	No
Education	No
Endocrine and metabolic disorders	No
Eye disorders	No
General interest	No
Genetics	No
Health inequalities/health equity	No
Infections and infestations	No
International development	No

Mental health and behavioural conditions	No
Musculoskeletal	No
Neurological	No
Nursing	No
Obstetrics and gynaecology	No
Oral health	No
Palliative care	No
Perioperative care	No
Physiotherapy	No
Pregnancy and childbirth	No
Public health (including social determinants of health)	No
Rehabilitation	No
Respiratory disorders	No
Service delivery	No
Skin disorders	No
Social care	No
Surgery	No
Tropical Medicine	No
Urological	No
Wounds, injuries and accidents	No
Violence and abuse	No

31. Language.

Select each language individually to add it to the list below, use the bin icon to remove any added in error.

English

There is not an English language summary

32. * Country.

Select the country in which the review is being carried out. For multi-national collaborations select all the countries involved.

China

33. Other registration details.

Name any other organisation where the systematic review title or protocol is registered (e.g. Campbell, or The Joanna Briggs Institute) together with any unique identification number assigned by them.
If extracted data will be stored and made available through a repository such as the Systematic Review Data Repository (SRDR), details and a link should be included here. If none, leave blank.

34. Reference and/or URL for published protocol.

If the protocol for this review is published provide details (authors, title and journal details, preferably in Vancouver format)

No I do not make this file publicly available until the review is complete

35. Dissemination plans.

Do you intend to publish the review on completion?

Yes

36. Keywords.

Give words or phrases that best describe the review. Separate keywords with a semicolon or new line. Keywords help PROSPERO users find your review (keywords do not appear in the public record but are included in searches). Be as specific and precise as possible. Avoid acronyms and abbreviations unless these are in wide use.

meta-analysis; bladder cancer; circulating tumor cells; prognosis; survival

37. Details of any existing review of the same topic by the same authors.

If you are registering an update of an existing review give details of the earlier versions and include a full bibliographic reference, if available.

38. * Current review status.

Update review status when the review is completed and when it is published.
New registrations must be ongoing so this field is not editable for initial submission.

Review_Completed_not_published

39. Any additional information.

Provide any other information relevant to the registration of this review.

40. Details of final report/publication(s) or preprints if available.

Leave empty until publication details are available OR you have a link to a preprint (NOTE: this field is not editable for initial submission).
List authors, title and journal details preferably in Vancouver format.
